# Spherical frame projections for visualising joint range of motion, and a complementary method to capture mobility data

**DOI:** 10.1111/joa.13717

**Published:** 2022-07-12

**Authors:** Eva C. Herbst, Enrico A. Eberhard, John R. Hutchinson, Christopher T. Richards

**Affiliations:** ^1^ Palaeontological Institute and Museum University of Zurich Zurich Switzerland; ^2^ Structure and Motion Laboratory Royal Veterinary College London UK

**Keywords:** joint mobility, movement visualisation, range of motion, spherical frame projection

## Abstract

Quantifying joint range of motion (RoM), the reachable poses at a joint, has many applications in research and clinical care. Joint RoM measurements can be used to investigate the link between form and function in extant and extinct animals, to diagnose musculoskeletal disorders and injuries or monitor rehabilitation progress. However, it is difficult to visually demonstrate how the rotations of the joint axes interact to produce joint positions. Here, we introduce the spherical frame projection (SFP), which is a novel 3D visualisation technique, paired with a complementary data collection approach. SFP visualisations are intuitive to interpret in relation to the joint anatomy because they ‘trace’ the motion of the coordinate system of the distal bone at a joint relative to the proximal bone. Furthermore, SFP visualisations incorporate the interactions of degrees of freedom, which is imperative to capture the full joint RoM. For the collection of such joint RoM data, we designed a rig using conventional motion capture systems, including live audio‐visual feedback on torques and sampled poses. Thus, we propose that our visualisation and data collection approach can be adapted for wide use in the study of joint function.

## Graphical abstract

Here, we introduce the spherical frame projection (SFP), which is a novel technique for 3D rotational joint motion, paired with a complementary data collection approach. SFP visualisations are intuitive to interpret in relation to the joint anatomy because they ‘trace’ the motion of the coordinate system of the distal bone at a joint relative to the proximal bone. Furthermore, SFP visualisations incorporate the interactions of degrees of freedom, which is imperative to capture the full joint RoM.

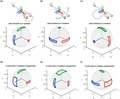

## 1 INTRODUCTION

Joint range of motion (RoM) is crucial for locomotor ability; it determines not only the limits of motion in a direction but also the number of possible directions (degrees of freedom; DoF). Because joint RoM is the space of possible joint orientations, it ultimately governs the reachable workspace of a limb; or ‘mobility’. For example, the many DoFs of frog hindlimbs enable frogs to modulate vertical and horizontal jump angles (Kargo et al., 2002). More generally, changes in RoM are thought to be important evolutionary innovations enabling various locomotor transitions in vertebrate history. For example, increased mobility in the elbow/knee, and wrist/ankle joints (relative to fish fins) enabled early tetrapods to bear weight on land with their limbs (Clack, 2012). Note that our study only considers rotational DoF (e.g. a three DoF joint means three rotational degrees of freedom, which may have additional translational degrees of freedom—see Manafzadeh and Gatesy (2021)).

Despite the biomechanical importance of RoM, experimentalists and theorists commonly rely on overly simple metrics such as a scalar range of angle limits (Hutson & Hutson, 2012; Pierce et al., 2012). Although simple measures are appropriate for one DoF joints, they cannot describe the bounds of three DoF joints (Brocklehurst et al., 2022; Kambic, Roberts, et al., 2017; Manafzadeh et al., 2021) and fail to capture interactions among individual DoF (e.g. the range along one DoF depending on the position of another). Hence, distinguishing between reachable versus unreachable joint space requires higher‐dimensional boundaries, rather than scalar limits. Furthermore, some joints may appear to operate principally in one DoF (e.g. flexion/extension of the knee), yet actually require additional DoF(s) to accurately describe their motion (Blankevoort et al., 1988; Kambic et al., 2014; Kargo et al., 2002; Manafzadeh et al., 2021). Therefore, a generalised, quantitative representation of RoM for three DoF joints is broadly required in the field of biomechanics.

### 1.1 Range of motion visualisation approaches

The central challenge to understanding three DoF joint RoM is in visualising a boundary between reachable and unreachable poses. Reachable poses may be illustrated as a projected 2D shape (spherical projection) or a 3D volume (volumetric RoM). Regardless of approach, an ideal visualisation method should meet three criteria: it is intuitive to define, intuitive to interpret and fully encapsulates 3D orientation through time. Without delving into mathematical details which are beyond the present scope, we discuss below how current spherical projection and volumetric approaches satisfy some, but not all, of the above criteria.

Volumetric RoM or joint mobility represents reachable joint poses as a 3D point cloud, around which a boundary surface or ‘hull’ is constructed. In one approach, the point cloud is created by Euler angle components (Euler space: Kambic, Roberts, et al. (2017), Manafzadeh and Padian (2018), and Richards et al. (2021)). Alternatively, the point cloud is from the vector components of quaternions (quaternion field space: Herda et al., 2003). Though the Euler space is intuitive to create, it is challenging to interpret; large Euler angle differences do not necessarily indicate a large physical distance between poses (although see Manafzadeh and Gatesy (2020) for a mathematical correction to this issue). On the other hand, visualising quaternion field space avoids the distortion of Euler spaces, but remains unintuitive to interpret. Further to the above disadvantages, the boundary surface for volumetric approaches is not straightforward to compute.

Alternatively, spherical projections illustrate reachable joint poses as points on the surface of a unit sphere, around which a boundary polygon can be more easily constructed by a variety of techniques (Chan, 2007; Korein, 1985; Wilhelms & Gelder, 2001). Most simply, the pose of a bone is represented by a vector, which is both intuitive to define and to interpret; however, a single vector is an incomplete description of orientation, as it neglects any information on long‐axis rotation or ‘twist’ (although see Baerlocher and Boulic (2001) and Wilhelms and Gelder (2001) for augmentations of the technique).

In summary, while volumetric representations fully describe 3D RoM, the visualisation is either unintuitive or distorts rotational distance. Additionally, computing a sensible boundary ‘hull’ enclosing a point cloud is challenging. Conversely, spherical projections of 3D vectors are straightforward to compute and visualise but are incomplete representations of RoM.

Combining the advantages of volumetric and projective techniques, we present a novel method to visualise 3D RoM data, termed the spherical frame projection (SFP) method. SFPs are intuitive to produce and interpret in relation to the joint anatomy because they ‘trace’ the motion of a joint. Moreover, they incorporate interaction among DoFs, which is imperative for characterising 3D joint function.

We also developed a new data collection methodology to capture detailed joint pose datasets including the interaction of degrees of freedom. This experimental setup includes live audio‐visual feedback on torques and sampled poses; the latter are visualised on spheres to enable the researcher to ensure full coverage of the pose space. Our methods are in three parts: (1) Mathematical formulation and graphical interpretation, (2) Experimental data collection and (3) Data processing.

## 2 MATERIALS AND METHODS

### 2.1 The spherical frame projection: mathematical formulation and graphical interpretation

A joint can be understood as the movement of the distal bone(s) relative to the proximal bone. The orientation of each bone can be characterised by a coordinate system, referred to here as an anatomical coordinate system (ACS), consisting of a set of orthogonal unit length axes X, Y and Z, which we refer to as frame axes. We introduce the SFP as a graphical representation that traces the movement of the ACS of the distal bone(s) relative to the ACS of the proximal bone at the joint. At the null pose (joint angles = 0), the axes of the distal ACS align with the proximal ACS (i.e. X, Y and Z). Rotation of the joint about the three axes causes reorientation of the distal ACS such that X, Y and Z endpoints are in a new position relative to the null pose. Directly rendering the X, Y and Z frame axes is an intuitive way to visualise any given orientation. We propose, therefore, to use the X, Y and Z frame axes directly in an RoM representation.

To understand how to interpret the SFP, imagine first a unit sphere originating at the joint centre which represents all possible movements of an unconstrained rotational joint. At the centre of this sphere is a frame of X, Y and Z axes which rotate with the joint. The null (reference or zero) pose axes are drawn in the middle of the sphere to provide a reference coordinate system. If each frame axis were allowed to draw on the surface of the sphere, the resulting pattern would indicate the 3D rotation of the ACS. Mathematically, the displacements of the three axes (due to rotation) are projected onto the sphere in a given sampling of RoM. The result is a distribution of points on the surface of the sphere which is divided into a triplet of regions; one for each frame axis. The reachable area of each frame axis is represented by the respective points, and a 2D surface boundary can be drawn around these points. Each frame axis can then be imagined to be constrained by its respective spherical surface boundary, thus creating an intuitive graphical representation of 3D joint RoM.

The rotation of the ACS is projected onto a sphere; an SFP. While the range of excursion of a single vector cannot fully represent 3D RoM, the rotation of the entire coordinate system can. Feasible rotation around any given frame axis will be constrained by the polygons traced by the other two axes. The projected shapes are three combinatorial pairs showing the interactions between the rotational DoF. The spherical frame projection is actually a visualisation of the rotation matrix itself because each row of the rotation matrix defines the endpoints of axes of the distal ACS relative to the proximal ACS (similar approaches have been previously implemented to visualise RNA helix orientations, see Bailor et al. (2011)). For an RoM dataset of rotation matrices, plotting the row of each matrix as a point coordinate gives three clusters of points on a sphere. Then, a boundary polygon can be defined either manually or statistically to fit around each cluster, for example using the reach cone algorithm of Wilhelms and Gelder (2001). Therefore, if orientations are already defined as rotation matrices, no additional conversion is required to produce the SFP. Even if not, the rotation matrix is so ubiquitous that any conversion will likely be straightforward, efficient and unambiguous in many common software packages.

To demonstrate how the spherical frame projection might be presented and interpreted, various examples are given below.

#### 2.1.1 Spherical frame projection: 1 DoF examples

**FIGURE 1:**
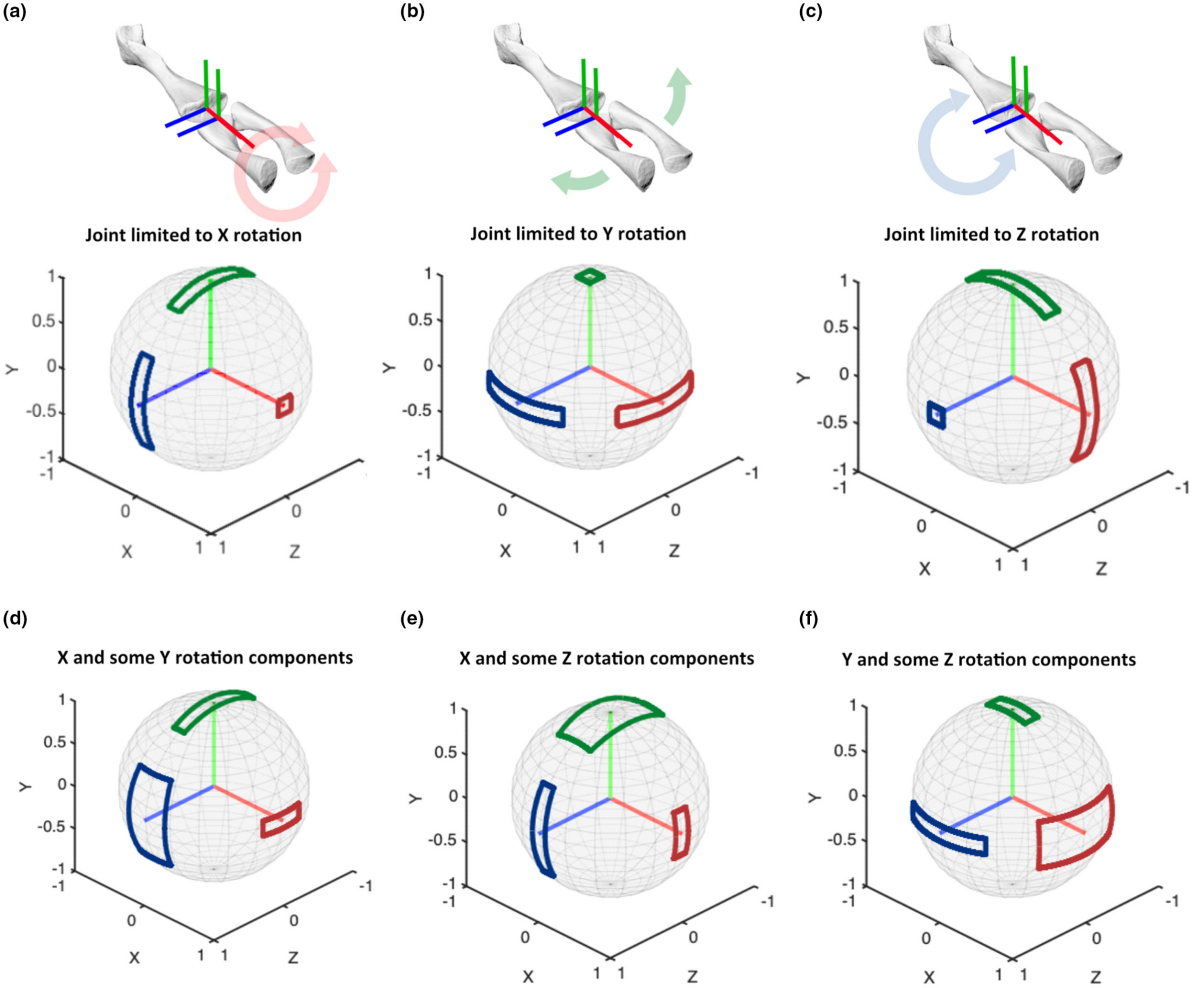
Example SFPs (spherical frame projections) demonstrating 1 DoF cases (a–c) and 2 DoF cases (d–f). The 1 DoF cases are accompanied by an example joint (salamander right knee joint), oriented such that flexion is positive rotation about the blue axis. The salamander joint provides an example of anatomical motions that can be depicted by the SFPs; here, the flexion/extension (FE) axis is Z, the abduction/adduction (ABAD) axis is Y, and the long‐axis rotation (LAR) axis is X, such that (a) shows LAR, (b) shows ABAD and (c) shows FE. In the 2 DoF joints (d–f), there are independent DoF ranges of ±30° and ±15° around the first and second listed axes; e.g. (d) has a range around X (red vector) of ±30°, visible in the ‘height’ of the blue and green polygons, and a range around Y (green vector) of ±15°, visible in the ‘width’ of the blue and red polygons. The range around each axis is described by a pair of polygons. Axis labels indicate lengths of reference frame vectors (e.g. 1 = unit vector length).

Hypothetically, the simplest case of RoM is a 1 DoF joint that can rotate only around one principal axis. Figure 1a–c shows how the boundaries of a spherical frame projection of RoM would appear for these simple 1 DoF joints.

This 1 DoF example most clearly shows how spherical frame projection represents the range of motion; the axes of the ACS are depicted inside a sphere and the movement of their endpoints on the surface of that sphere are restricted by imaginary polygons. In this trivial case, the polygons are lines with no width. One can then interpret spherical frame projections by imagining a rotation about the red, green or blue frame axes in a way that keeps each colour within its respective boundary walls. Some rotations are not possible because the frame axes leave their respective boundaries.

For the purpose of demonstration, we have illustrated Figure 1a–c with a range of ±30° rotation around a single frame axis. Note that the excursion of the rotation axis itself is fixed with regard to the endpoint on the sphere; it is tightly bounded by its polygon, which in this case is the smallest possible polygon enclosing the tip of the rotation axis. The remaining two axes move equally along a common arc around the rotational axis, and their bounding polygons have a long side with an arc of 60° representing the ±30° range. We note that the polygons in this 1 DoF case are actually lines with no width because the axes tips can only move along a single arc; the polygon widths in Figure 1a‐c are just for illustrative purposes.

#### 2.1.2 Spherical frame projection: 2 DoF examples

Although the above 1 DoF case is trivial and does not need a 3D representation, it nevertheless demonstrates that two polygons share common information (Figure 1a–c). This property becomes more evident when introducing a second degree of freedom.

In our example, a 3 DoF ball joint will be constrained to produce a 2 DoF joint. For demonstration, we assign the first DoF to have ±30° range around a principal frame axis, and the second joint will have ±15° range around the second axis.

In an Euler space, our hypothetical joint would appear as one rectangular RoM with side lengths of 60° and 30° for the first and second axes, respectively. In the spherical frame projection, the same limits appear as three discrete rectangles on the surface of a sphere (Figure 1d–f). As in the 1 DoF case above, the polygons are rectangles, but in the 2 DoF case, their widths can vary. Each shape on the spherical frame projection represents the RoM of a pair of axes at any given point. So, if X and Y are the two main rotational axes, the Z polygon will have side lengths reflecting each respective range. Similarly, the Y polygon will show the X range on one side and the Z range on the other.

Three possible combinations of two ordered axes are shown in Figure 1d–f. Between them, the set of three projected rectangles are all the same but appear in different locations and rotations. Figure 1 is therefore a convenient reference for understanding how each polygon shows the interactions of two DoF.

#### 2.1.3 Spherical frame projection: 3 DoF examples

The above examples are for demonstration purposes only; in practice, spherical frame projection is not necessary for 1 DoF and 2 DoF joints, whose RoM is more easily shown by traditional graphs. However, as we illustrate below, the strength of the spherical frame projection is that the range of motion is not defined by the rotation around a frame axis, but rather by the excursion of the frame axes themselves. Hence, because the spherical frame projection displays the excursions of each axis due to rotation about other axes, the spherical frame projection becomes particularly useful in 3 DoF cases.

Consider, for example, an Euler sequence in which Z is assigned as the flexion/extension axis (e.g. Arnold et al., 2014; Kambic, Roberts, et al., 2017; Manafzadeh et al., 2021; Manafzadeh & Padian, 2018; Nyakatura et al., 2019). Although this axis assignment may work well for some poses, ambiguities may arise in other poses. Using conventional visualisation approaches, the contributions of rotations about X and Y axes could mask the effects of large Z rotations, causing the joint to appear less flexed. For example, starting from the null pose, clockwise rotation about the Z‐axis rotates the X‐axis vector up. For our example, we will refer to this as flexion (as in Figure 1), although determining flexion/extension directions is arbitrary and can vary with different studies. However, once the ACS is rotated, for example by 90 around the Z‐axis, subsequent rotation around Y will rotate the X‐axis back ‘downwards’ towards the horizontal plane, cancelling the visual effect of the initial Z rotation. While the final pose would have a flexion angle of 90^°^ in Euler terms, the direction of the X vector could be anywhere below or above the horizon.

The spherical frame projection solves the above problem in that each polygon unambiguously defines the allowable movement of each axis vector. For the Z vector, the horizontal and vertical dimensions of the polygon on the sphere consistently define adduction/adduction and long‐axis rotations, respectively (depending on joint anatomy and convention), regardless of the history of rotations around specific axes leading to a final pose.

Hence, three polygons are necessary for SFP because two alone would leave the motion of one axis ambiguously defined. If, for example, only the Y and Z polygons are defined, the X vector is implicitly limited in some way because it must be orthogonal to the Y and Z vectors. However, if the space of the Z polygon travels far enough in one direction that the X‐axis can be near the null pose of the Z‐axis, then the X‐axis ‘inherits’ the range defined by the Y polygon. We now describe what happens in two cases, one in which two degrees of freedom are explicitly defined by polygons on the SFP and another in which all three degrees of freedom are explicitly defined.

**FIGURE 2:**
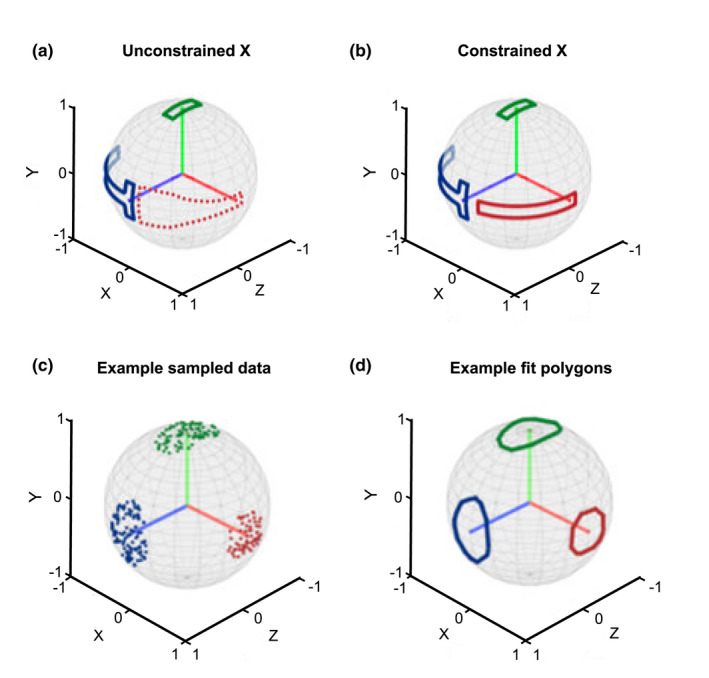
(a, b) Demonstration of why three spherical polygons are necessary to describe any non‐trivial 3D RoM. (b) Hard constraints are defined only for Y (green) and Z (blue) frame axes. The range of the X (red) vector is implicitly bound as the space of cross‐products of all orthogonal feasible Y and Z (red dotted area). Rotation around Z is limited in the null pose, but more free after a large rotation around Y. (b) X is additionally constrained within the implicitly limited boundary, leading to a different overall RoM (rotation around Z is limited in all cases). (c) Example 3 DoF joint data with the generated dataset. The coordinates of the points on the sphere each correspond to one row of one rotation matrix in an orientation dataset of many rotation matrices. (d) Polygons are automatically fit around the point data in (c), illustrating the bounds of the range of motion. Axis labels indicate lengths of reference frame vectors (e.g. 1 = unit vector length).

Figure 2a shows a case in which the Y and Z polygons are defined explicitly; the X polygon shape is based on all possible X‐axis positions given the pre‐defined positions of the Y and Z axes (since the X, Y and Z axes are orthogonal). At the null pose, flexion/extension (rotation about Z) is restricted, but after counterclockwise rotation about the Y‐axis, more flexion/extension becomes possible.

Figure 2b shows a case in which the X positions are explicitly defined such that regardless of the rotations about the Y‐axis, rotation about the Z‐axis (flexion/extension) is not possible.

### 2.2 Experimental measurement of RoM

To evaluate the effectiveness of the new spherical frame projection of RoM in the context of anatomical data, we designed a novel rig using motion capture. Our companion paper on the joint RoM of *Salamandra salamandra* is a case study of these methods (Herbst et al. 2022). The general setup of the data collection and data transformation is outlined below; these new methods can be applied to a variety of organisms because the rig components can easily be scaled up or down. The main steps include visual kinematic data capture and then transforming data from the motion capture markers to the joint. Researchers who already have joint‐centric data (e.g. from XROMM analysis: Brainerd et al., 2010) can directly create the SFP with a rotation matrix of the distal ACS relative to the proximal one, as obtained from the Euler angle output of the oRel XROMM_mayaTools shelf tool.

#### 2.2.1 Visual kinematic capture

The primary goal of RoM experiments is to measure the joint orientation at all reachable poses. Measuring joint RoM about separate axes in isolation (as in e.g. Haines (1942), Hutson and Hutson (2014), and Kargo et al. (2002)) does not truly capture the joint RoM, since the position of one axis can affect the rotations about other axes (Kambic et al., 2014; Kambic, Biewener, et al., 2017; Kambic, Roberts, et al., 2017; Manafzadeh & Padian, 2018). Therefore, explored poses should cover the interactions of rotational axes, and measurements should be in one of the fully representative 3D rotational formats.

Many solutions exist to measure 3D joint pose, of which XROMM (Brainerd et al., 2010; Manafzadeh, 2020) has become popular in comparative biology (e.g. Arnold et al., 2014; Kambic, Roberts, et al., 2017; Manafzadeh et al., 2021; Manafzadeh & Padian, 2018; Nyakatura et al., 2019). The advantage of XROMM is direct access to relative bone movements both in vivo and ex vivo. However, the x‐ray radiation involved makes manipulation of post‐mortem joint specimens difficult; capture periods and therefore sampled joint poses can be limited by radiation exposure thresholds. Furthermore, the experimenter must be far removed from the capture volume and therefore has no fine control of the joint movement, which is especially problematic for small specimens.

Capture period, and equivalently pose sample size, is an important factor because every instantaneous joint pose represents a single point in a high‐dimensional volume of possible orientations. While it is impossible to capture the infinite subset of points within the reachable space, a larger sample better approximates the true RoM. The ability to finely control the joint as RoM is explored is critical for a similar reason. Samples that are well and evenly distributed are preferable to random sampling. Additionally, lack of fine control could lead to joint failure where too much force is applied.

For our companion paper (Herbst et al. 2022), we developed a new experimental approach using visual kinematic capture using multiple cameras. We describe the foundations for the application of this approach in this paper, whereas the companion paper provides a case study with real biological data. Our method operates on a similar principle to XROMM, though using visible and near‐visible light removes the capture period limitation. We used the Qualisys photometric motion capture system (Qualisys AB, Göteborg, Sweden). The system uses specially calibrated cameras sensitive to infra‐red (IR) light to track the position of IR reflective markers.

The commercial software package Qualisys Track Manager (QTM), provides utilities for calibrating, capturing and processing kinematic data. If the position and orientation of the cameras are known, then the 2D position of a reflective point on the image sensor of two or more cameras is enough to resolve the point coordinate in 3D space. Six Qualisys Opus cameras were employed around the experiment table. The cameras were spaced at roughly 50° intervals around the axis normal to the table, alternating steep and shallow viewing angles with respect to the table plane. In this manner, points could be robustly tracked within the capture volume even when occluded from some viewing angles. The spacing additionally provided an access point for the operator of the experiment.

#### 2.2.2 Measuring torques

Direct measurement of joint torque (the passive torque required to achieve a given joint pose; e.g. Molnar et al., 2014) could make the determination of RoM limits more objective and repeatable. All RoM studies cited above have assessed RoM limits subjectively; an experimenter manipulated joints until a certain, qualitative, resistance was felt. This is probably adequate in large, robust joints and where limits are generally osteological (from bone on bone contact). In these cases, the passive joint torques at RoM limits can be very high and are easily noticeable.

However, joints are also limited by the stretching or apposition of soft tissues including muscle, fascia, ligaments and the joint capsule (Clarkson, 2000; Molnar et al., 2014). While bone contacts can lead to sharp boundary conditions, soft tissues exhibit non‐linear and at times elastic stress–strain relationships (Fung, 1967; Lieber et al., 1991).

Any material will have a structural failure under a high enough force or torque. When passive joint torques are composite, the force limit of some elements may be much lower than others. In other words, advancing the joint up to a sharp osteological boundary may have already caused the failure of tissue elements. For smaller and more delicate joints, and where passive tissues are involved, more care needs to be taken around joint boundaries. Measuring torque about the joint and providing real‐time feedback during a trial can help a researcher explore RoM boundaries within a safe and fixed threshold of applied torque.

To measure the passive constraint torque of the joint at any position, our experimental setup included a force‐torque transducer. This sensor was an ATI Nano17 6‐axis force and torque (F/T) transducer. The sensor has specially designed titanium bridges as an internal structure, with strain‐sensitive resistors across the bridges. External forces deform the stiff bridges minutely and cause a change in resistance depending on the magnitude and direction of the applied force. With a factory‐supplied calibration matrix, the voltage measured across the resistors can be converted into two independent vectors of force and torque relative to the XYZ coordinate system of the sensor.

The Nano17 was connected to an ATI IFPS (Interface and Power Supply) box, which powers and amplifies sensor voltages to six channels in a ±10 V range. These channels and a common ground signal were supplied into a National Instruments DAQ (Data Acquisition Device), which had a USB connection to the host laptop.

#### 2.2.3 Joint rig

A special rig was devised to measure the relative displacement and force between any two body segments. It consisted of a fixed body (referred to as the base) and a moving component (referred to as the handle component), connected to each other by the joint anatomy. The frame (coordinate system) of the base was designated as the global coordinate system. The base held a force sensor and the proximal bone of the joint, while the handle component had trackable markers and the distal bone(s) of the joint. The absolute motion of the markers was then directly associable with the relative joint movement. Similarly, the absolute forces and torques measured on the base frame could be associated with joint‐centric torques. A schematic of the rig is shown in Figure S1.

These associations between rig measurements and joint properties rely on the rigid connection between the rig and the anatomy around the joint. At the same time, the experimental setup should allow for the mounted anatomy to be changed across trials and specimens—in other words, a single joint should not be permanently affixed to the entire system. All of the rig parts (base, force sensor, proximal bone, distal bone and markers) had their own frames (coordinate systems), as did removable small plates connecting the bones to the rig, which enabled the motion capture and force data to be transformed to the joint. Further details on the construction of the joint rig are given in the Appendix.

#### 2.2.4 Real‐time audio‐visual interface to guide experiments

A MATLAB function class (see Data Availability section) was written to provide real‐time feedback to the experiment operator and automate as much of the trial procedure as possible. The class constructor initiated a connection with the DAQ over USB and set up a transmission control protocol link with QTM. It also opened a graphical user interface (GUI) in a new window on the laptop. Aside from determining and displaying the sequences of the trial procedure, the GUI also provided visual and audio feedback on joint kinematics and forces.

Joint orientations were streamed from QTM, and converted and rendered on two spherical plots. One plot showed the position of the distal bone as a point on a sphere, where the point represented the long axis of the handle, which is roughly aligned with the X (long axis) of the distal bone, thereby capturing flexion/extension and abduction/adduction. The other showed a point as the vector component of the pose quaternion, normalised to unit length. As the vector encoded a rotational axis with a length related to the angle around that axis, normalising the vector left only the rotation axis information. Therefore, the second sphere displayed information on long‐axis rotation.

In addition to showing the current pose, both spheres showed heatmaps of coverage. Moving the pose point through a region on each sphere left a trace of a higher intensity colour, which became the highest intensity after three independent passes. Orientation coverage could therefore be gradually ‘painted on’ by the manipulations of the experimenter, easily revealing any obvious gaps in coverage.

The three components of joint torques were shown on a separate plot as a moving point, increasing in size with magnitude. Additionally, an audible tone was generated based on torque magnitude; for any torque above a cut‐off threshold, the tone would play and increase in pitch up to a defined upper torque limit.

The F/T sensor measured forces and torques relative to its own sensing frame; forces on the joint would result in measured torques not actually present in the joint, due to the offset of the adapter plate. The joint‐centric torques were calculated based on a manually pre‐determined estimate of joint displacement and orientation. This process for frame conversion is more fully explained below.

The MATLAB class also provided buttons to control the data collection. Recordings of kinematics and torques could be started, paused and stopped and then saved in a bundled format with automatically generated filenames. Providing real‐time software support enabled more efficient, effective and objective data collection.

#### 2.2.5 Data collection recommendations

As previously mentioned, ensuring even coverage of the whole reachable space and avoiding failure of passive tissue structures are key targets in RoM studies. We, therefore, recommend decomposing measurements of each specimen into multiple trials. If torque recordings are used, the magnitude of torque load on the joint can be set beneath some maximum limit; then, the space of possible orientations would be swept fully, but prioritising range limits in a systematic manner. We recommend randomising the order of the sequences between trials to balance the bias and minimise tissue strain (see Herbst et al. 2022).

The visual indication of pose coverage can be used to keep track of progress in each trial and to check for any changes in range, while the audio signal for joint torque magnitude indicates where limits were reached.

After all trials are concluded, the proximal and distal bones of the joint should be separated, leaving two sets of bones along with their mounting plates. These bones should then be scanned with the plates to determine the configurations of the bones during the experiments (see section 2.3.1).

### 2.3 Data processing

#### 2.3.1 Joint orientation from motion capture marker positions

The final data desired from the experiment to visualise RoM are the set of orientation samples of the ACS of the distal bone expressed in the proximal ACS. This meant transforming the orientations of the handle frame (based on the positions of the Qualisys markers) with respect to the base (global) frame into orientations of the distal bone with respect to the proximal bone. Note that when using SFPs with other datasets, such as XROMM data, where the relative bone movements are also known, the following transformation steps are not necessary.

**FIGURE 3:**
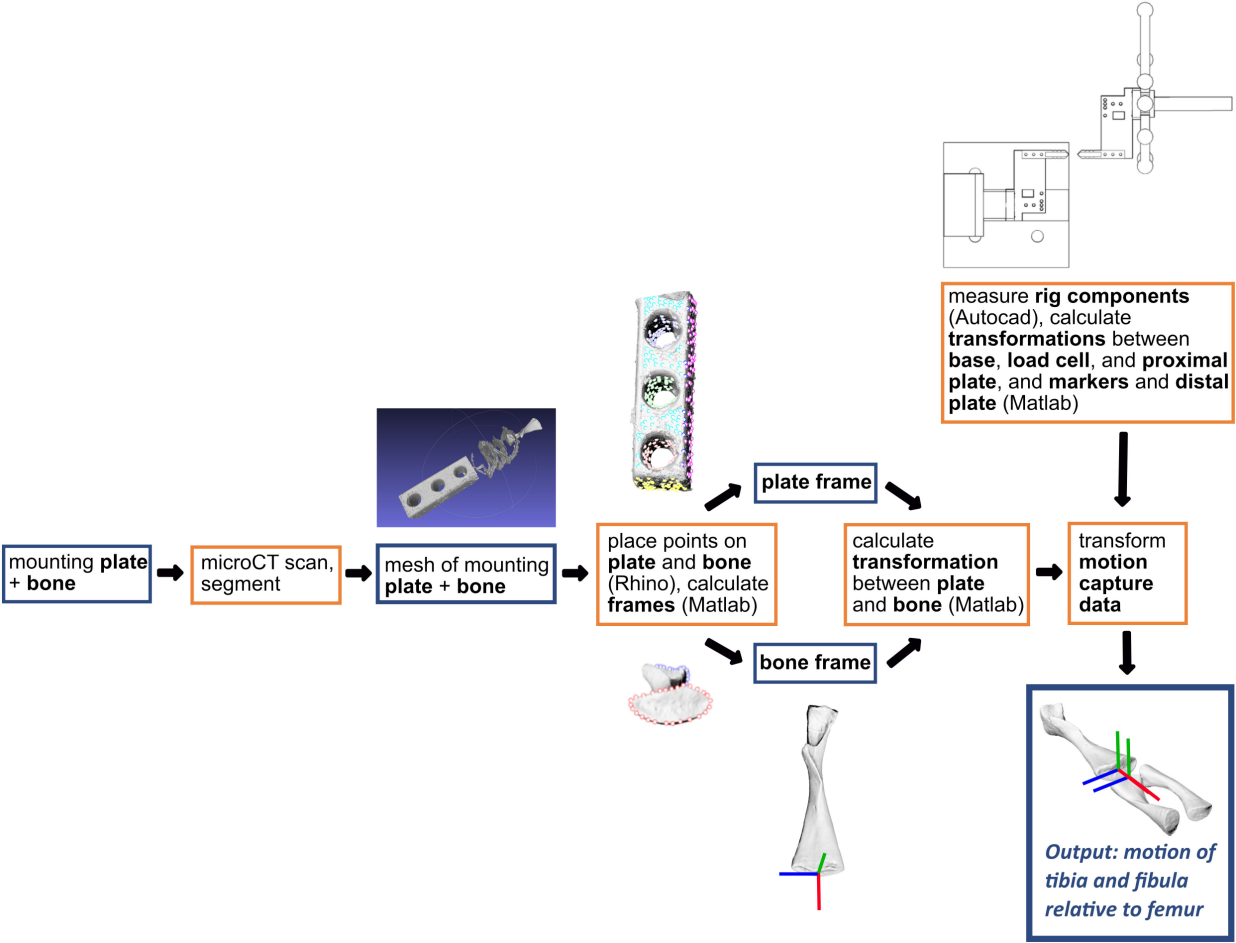
Workflow example with a salamander knee joint demonstrating how the Qualisys motion capture data is transformed into an anatomically meaningful (i.e. joint‐based) coordinate system. Blue boxes indicate objects, orange boxes indicate processes.

The alignment of the distal bone with respect to the proximal could be found through a series of linear transformations, some of which are fixed and some of which vary between specimens (Figure 3). The initial data were the positions and orientations of the markers on the distal segment through time with respect to the base frame (e.g. the global reference frame). We denote this as a homogeneous transformation matrix ^markers^ *T*_base_ ∈ *R* ^4*×*4^. The transformation to be derived was that of the distal bone ACS (dACS) with respect to the proximal bone ACS (pACS), ^dACS^ *T*_pACS_. First, the location of the proximal ACS in the base frame was calculated from the specimen‐specific pose of the proximal bone on the proximal mounting plate (pPlate) and the known rigid transformation of the plate in the base frame:

$${}^{\text{pACS}}T_{\text{base}}{=}^{\text{pACS}}T_{\text{pPlate}}\times^{\text{pPlate}}T_{\text{base}}$$

The distal ACS similarly had a specimen‐specific location on the distal mounting plate (dPlate), which in turn has a known rigid transformation with respect to the markers. This allowed the distal ACS to be expressed in the marker frame:

$${}^{\text{dACS}}T_{\text{markers}}{=}^{\text{dACS}}T_{\text{dPlate}}\times^{\text{dPlate}}T_{\text{markers}}$$

The transpose of a homogeneous transformation matrix (denoted by *T*) gives its inverse, such that ^base^ *T*_pACS_ = ^pACS^ *T*_base_^*T*^. From these matrices, the desired relation could be calculated:

$${}^{\text{dACS}}T_{\text{pACS}}{=}^{\text{dACS}}T_{\text{markers}}\times^{\text{markers}}T_{\text{base}}\times^{\text{base}}T_{\text{pACS}}$$

The orientation data for the SFP were then just the rotational component of the transformation matrix, ^dACS^ *R*_pACS_ ∈ *R* ^3*×*3^.

The marker frame relative to the base frame ^markers^T_base_ was the time‐varying data captured from the Qualisys kinematics, and all remaining transformations except the bone orientations relative to their respective mounting plates were constants of the joint rig, which was constructed to known dimensions.

The frames of the bone segments relative to their respective mounting plates ^pACS^ *T*_pPlate_ and ^dACS^ *T*_dPlate_ were unknown at the time of the trial and differ between specimens as a result of the manual glueing and binding procedure. To precisely measure the mounting configuration of each bone, *μ*CT scans were used. By terminating the trials of each specimen with full joint separation, it was possible to individually scan the distal and proximal segments of each joint still mounted to the acrylic plates. By including the acrylic plates in the scan, the relative relationship between the bone segment and the mounting plate was preserved. To find the transformation from bone frame to mounting plate frame, we assigned coordinate systems for the mounting plate and bone (Figure 3). Details of assigning coordinate systems and transforming the data are given below.

#### 2.3.2 Mounting plate frame orientation

The mounting plate is a manufactured object made with known dimensions and properties. We defined a local frame with axes parallel to the edges of the plate. The origin was arbitrarily chosen to be the ‘top‐right‐ back’ corner. To find the local frame for the mounting plate in the arbitrary scan orientation, we placed points using Rhinoceros 3D v5.4 (Robert McNeel & Associates), a 3D graphics and computer‐aided design (CAD) programme. In theory, only three noncollinear points are needed to identify the pose of a 3D body when the exact relation between the points is known. However, using many points eliminates the need for precise relative placement, and serves to filter noise in the plate material, scan digitisation or point placement. We placed points on faces, edges and screw holes of the plate, and then used Matlab to calculate planes and axes to then calculate the plate coordinate system from these points (Figure 3). While generally orthogonal, the three resultant axes were fully and formally orthogonalised by a recursive averaging of cross‐products that attracted or repelled each axis marginally in turn until the dot products of all combinations were 0.

#### 2.3.3 Bone orientation

Finding a local coordinate frame for the bone is more difficult than for the plate because of its organic shape; not only are there no naturally orthogonal and linear features, but the exact dimensions and relations of features change between specimens. There have been many efforts in previous literature to define bone coordinate systems from skeletal features. While a standard has been developed for humans (Wu et al., 2002), comparative vertebrate biology faces the difficulties of having largely different morphologies between species, changing not just features on a given bone but also the postural context of a given joint relationship Gatesy et al. (2022).

The solution to the problem generally depends on the nature of the joint. Geometric primitives are often fitted to the joint morphology; for example, in bicondylar joints such as the knee, a cylinder can be fit to the condyle to define a principle axis (e.g. Bishop et al., 2021; Gatesy et al., 2022; Kambic et al., 2014; Kambic, Roberts, et al., 2017; Manafzadeh & Padian, 2018; Miranda et al., 2010). Another method is to calculate the principal inertial axes of the bone using the scan data (Crisco & McGovern, 1997; Kambic et al., 2014), of which the least inertial axis runs along the shaft of long leg bones.

For the salamander joints used in our companion paper (Herbst et al. 2022), a contrastingly simple but sufficiently repeatable method was used. Our method worked on specimens such as salamanders where geometric primitives cannot easily be fit into the morphology of some bones. In Rhinoceros software, points were evenly placed along the proximal and distal edges of each bone (Figure 3), and lines were fit through these points. The salamander‐specific coordinate systems are described in detail in our companion paper (Herbst et al. 2022). Our sensitivity studies showed good agreement with other methods and repeatability between users.

#### 2.3.4 Constant rig transformations

After determining the relative transformations between bones and mounting plates, the remaining transformations are either constant or directly measured by known values. The dimensions of the printed base and the handle containing the markers are known from the CAD‐modelled geometry. Distances in the real joint rig and plate components were measured with callipers to determine any shrinkage or tolerances in 3D printing and laser cutting, and an adjusted CAD model was created to reflect these values.

These relationships were additionally verified using the Qualisys cameras with the joint rig in various configurations; using the various mounting holes in the mounting and adapter plates, the marker tree was fixed to the base stand in various offsets and orientations, with and without certain mounting plates and adapter plates. The difference between each measurement of the marker tree frame with and without certain components was used to calculate the offsets and rotations resulting from each individual component in the joint rig.

#### 2.3.5 Data visualisation

We developed a custom MATLAB script to create the SFP visualisations. The method is documented in the Github repository (Github repository will be published upon acceptance). Briefly, the X, Y and Z frame axis endpoints are plotted as points on a sphere. The overall pose space polygons are created based on an interactive GUI where the user selects small patches based on whether they contain datapoints or not. The visualisation also enables plotting of various datasets on the same figure (e.g. in vivo and ex vivo data, see Herbst et al. 2022 for examples).

## 3 LIMITATIONS

While our approach can be used to visualise joint RoM, using the SFP data to explore viable joint poses can lead to false positives (i.e. poses that are not anatomically feasible). For example, if you create a spherical frame projection dataset and randomly manipulate the axes until all axis endpoints fall inside the polygons, this is not sufficient to check the viability of a pose. This is because in rare cases you could get X, Y and Z endpoints that are orthogonal but actually produced from separate joint poses (e.g. X‐axis position from one pose, Y‐axis from another, Z‐axis from another). This false positive could likely be avoided by starting out with a known viable pose and then moving the joint to the pose of interest along a path whose intermediate positions all stay within the polygons, but further testing is needed for such applications.

Furthermore, the SFP was created to capture rotational RoM. Further DoF (translation) could be layered onto the graphs. Although directly graphing translations (e.g. as translations in the graph) remain difficult because the translation corresponding to each frame endpoint would be challenging to keep track of, translation data could possibly be added to the SFP using colour gradients to represent ranges of translations.

## 4 CONCLUSION

Joint RoM is a vital component of locomotor biomechanics, as various behaviours are only accessible when limb joints are able to move to corresponding poses. More measurements and analyses of joint RoM across species would enable richer comparative studies relating morphology to behaviour. This drove our motivation to improve experimental methods for data collection and mathematical methods for data visualisation and analysis.

The SFP visualisation method enables an intuitive representation of the rotational joint range of motion including the interaction of degrees of freedom. We also designed a new method combining automatic kinematic capture with joint torque feedback with live joint torque feedback and live visual feedback showing the pose space sampled in the trial. In Herbst et al. (2022), we applied our novel methods to collect a detailed dataset of salamander hip and knee joint poses including interactions of degrees of freedom. The salamander poses are visualised using our new SFP visualisation method, demonstrating the effectiveness of the new data collection method in sampling a detailed pose space.

Overall, the visual capture proved to be a very viable method to record the orientations of a manipulated joint. Motion capture systems are more accessible than XROMM and compared to the XROMM methodology, the operator can be close to the joint and spend as long as necessary in manipulations. This is especially important for small specimens that could not be manipulated in an XROMM setup without too much radiation exposure to the researcher. The Qualisys system also provides the benefit of real‐time tracking conversions, so that the current orientation and previously sampled orientations can be visualised throughout the trials.

The component of feedback outside of subjective judgement is absent from other RoM investigations (e.g. Arnold et al., 2014; Hutson & Hutson, 2014; Kambic, Roberts, et al., 2017; Manafzadeh et al., 2021; Manafzadeh & Padian, 2018; Molnar et al., 2014). Measuring torque at the joint is helpful during the trial to make results more objective, repeatable and comparable. Additionally, systematic use of torque feedback can be used to maximise the data from joint specimens. The sensory data from the load cell can be transformed into passive joint torques, which could be used to enforce more accurate boundaries in a simulation, distinguishing between hard and soft limits. Future studies could present a full map of passive joint torques as a function of joint orientation. Furthermore, by a repetitive sampling of the same pose and measuring the torque, it would be possible to check if ligament stability deteriorated over time (i.e. if lower resistance occurred in later subtrials).

There is still room for improvement in the quality and repeatability of RoM experiments through further technological integration. Manual manipulation of joints is still the standard approach in RoM studies. With the introduction of a force sensor and real‐time feedback of joint pose and estimation of joint torques, it would be possible to perform a robot‐assisted search of joint space. A 6 DoF robot manipulator would be able to sweep the space of orientations much more precisely and repeatably than a human, and would also be able to respond more effectively and objectively to torque signals at boundaries.

## AUTHOR CONTRIBUTION

Eva C. Herbst and Chris T. Richards wrote the paper, based on the thesis chapter from Enrico A. Eberhard. Enrico A. Eberhard developed the SFP approach and wrote the code and curated the data. Enrico A. Eberhard, Eva C. Herbst and Chris T. Richards contributed to project conceptualization. Enrico A. Eberhard and Eva C. Herbst developed and tested the experimental rig and created the figures and curated the data and code. All authors edited the manuscript. John R. Hutchinson and Chris T. Richards acquired funding and supervised the project.

## FUNDING INFORMATION

This work was funded by a European Research Council Starting Grant PIPA338271 and the Natural Environment Research Council NE/K004751/1.

## CONFLICT OF INTEREST

The authors declare no competing interests.

## Supporting information


Figure S1

## Data Availability

The code for generating spherical frame projections can be found at doi.org/10.5281/zenodo.6791211 and the code for recording motion capture data with live feedback can be found at https://doi.org/10.5281/zenodo.6791217.

## Construction of the Joint Rig

To facilitate a rigid but easily detachable connection, the bone segments on either side of a joint were each affixed to a small removable mounting plate (Figure S1 segment D) using a combination of fine copper wire wrapping and two‐part epoxy resin (see Herbst et al. 2022 for figures of salamander joint rigging). The standard mounting plate and all other plates referenced in this section were cut from 5.3 mm acrylic sheets with a 60 W CO2 laser. The jointed end of each bone was kept at a distance from the mounting plate to prevent the plate or resin from interfering with the joint range of motion. To ensure rigidity of the connections, the ratio of bone on the plate to bone protruding on the joint side was generally 1:1.

The base comprised a stand, force sensor and offset plate (Figure S1). The stand was 3D printed from PLA filament and rigidly bolted to a table. The force sensor was mounted on the stand at a height of 115 mm with a small adapter plate. On the other side of the F/T sensor, another small adapter plate and offset plate provided a fixing point for the mounting plate.

The handle component comprised a handle, a ‘tree’ of infra‐red (IR) reflective markers and another offset plate. The tree was laser‐cut from 5 mm plywood and had 7 spherical IR markers at various offset lengths at 45^°^ increments around the centre. Each marker was given an ID number from 1 to 7, clockwise around the tree. Arranged in this manner, the distance between any two markers is unique; knowing the 3D coordinates of any three markers is enough to find the ID of each, and thereby reconstruct the entire marker tree. The specific configuration of the markers was programmed into the Qualisys Track Manager software (QTM) to return the position and orientation of the marker tree.

The purpose of the two offset plates was to maximise the free RoM of the rig. If the bone segments were mounted inline with the F/T sensor and marker tree, the relatively large marker tree would collide with the base.
