# Supplementary figures and images for: Spherical frame projections for visualising joint range of motion, and a complementary method to capture mobility data
Source: J Anat. 2022 Jul 12;241(4):1054–65. doi: 10.1111/joa.13717 (PMC9482700; doi:10.1111/joa.13717)

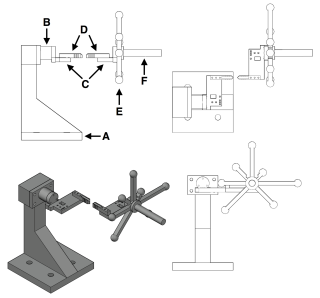

Supplement: Supplementary file 1 — Figure S1 [file JOA-241-1054-s001.tif]
